# Mating and longevity in ant males

**DOI:** 10.1002/ece3.2474

**Published:** 2016-11-23

**Authors:** Sina Metzler, Jürgen Heinze, Alexandra Schrempf

**Affiliations:** ^1^ Zoology/Evolutionary BiologyUniversity of RegensburgRegensburgGermany; ^2^ IST Austria (Institute of Science and Technology Austria)KlosterneuburgAustria

**Keywords:** *Cardiocondyla*, life span, reproduction, trade‐off

## Abstract

Across multicellular organisms, the costs of reproduction and self‐maintenance result in a life history trade‐off between fecundity and longevity. Queens of perennial social Hymenoptera are both highly fertile and long‐lived, and thus, this fundamental trade‐off is lacking. Whether social insect males similarly evade the fecundity/longevity trade‐off remains largely unstudied. Wingless males of the ant genus *Cardiocondyla* stay in their natal colonies throughout their relatively long lives and mate with multiple female sexuals. Here, we show that *Cardiocondyla obscurior* males that were allowed to mate with large numbers of female sexuals had a shortened life span compared to males that mated at a low frequency or virgin males. Although frequent mating negatively affects longevity, males clearly benefit from a “live fast, die young strategy” by inseminating as many female sexuals as possible at a cost to their own survival.

## 1 Introduction

Life history theory predicts the occurrence of trade‐offs between fitness traits whenever resources are limited. For example, food‐restricted individuals have to decide whether and how much to invest into current reproduction or maintenance (e.g., Chippindale, Leroi, Kim, & Rose, 1993). Insufficient investment into maintenance, for example, repair, immune function, and growth, entails reduced longevity and future reproduction, resulting in the prominent fecundity/longevity trade‐off (Stearns, 1992; Williams, 1966). The costs of reproduction are well documented for females of many species (see Schwenke, Lazzaro, & Wolfner, 2016), but data are more ambiguous for males (Kotiaho & Simmons, 2003; Olsson, Madsen, & Shine, 1997). At least in some species, males suffer reproductive costs, for example, from the production of sperm and seminal fluids (Pitnick, 1996; Van Voorhies, 1992), competition over access to females (Barnes & Partridge, 2003), and an increased immune defense against sexually transmitted pathogens (Schwenke, Lazzaro, & Wolfner, 2016).

Interestingly, queens of social insects (honeybees, termites, and ants) appear to lack the trade‐off on the individual level. Instead, egg‐laying rate and life span of queens may be positively associated (Heinze & Schrempf, 2008, 2012; Lopez‐Vaamonde et al., 2009), probably because the costs of reproduction are at least partly borne by the workers (Helanterä, 2016). Little is known about the association between fecundity and longevity in social insect males. While termite kings are both long‐lived and highly fertile (Hartke & Baer, 2011), reproduction obviously bears a high price in honeybee drones, in which mating is suicidal (Starr, 1984). How reproductive efforts and life span are associated in other social insect males remains largely unstudied, as mating often takes place on the wing and is difficult to observe (Heinze, 2016; Wilson & Hölldobler, 1990). This is different in the ant genus *Cardiocondyla,* where males regularly mate inside their maternal nests (Boomsma, Baer, & Heinze, 2005). Males therefore can monopolize mating with virgin queens and has led to an environmentally determined male diphenism with “typical” winged disperser males and locally competing wingless males. In most species, wingless males fight fiercely and try to exclude all their wingless rival males from mating (Heinze et al., 1993; Kinomura & Yamauchi, 1987). Wingless *Cardiocondyla* males are exceptional in that they produce sperm throughout their lives. While wingless *Cardiocondyla* males are therefore capable of mating with larger numbers of female sexuals (Heinze & Hölldobler, 1993), males of all other social Hymenoptera are sperm‐limited because their testes degenerate before or shortly after adult emergence (Hölldobler & Bartz, 1985). Therefore, wingless *Cardiocondyla* males are a suitable model to investigate the interrelation between reproduction and longevity in ant males. In contrast to queens, ant males do not actively contribute to the social life of the colony and also receive less care than the queens. We therefore hypothesize that, because males are less well integrated in the society, their costs of reproduction will not be borne fully by the workers and increased mating activities will therefore shorten their life span.

## 2 Material and Methods

We set up 115 experimental colonies by separating a mated queen, 15–20 workers, and a wingless male pupa from our laboratory stock colonies (collected in Ilhéus, Brazil) of the ant *Cardiocondyla obscurior* (Wheeler, 1929) in new nest boxes. The ants were kept in the laboratory under near‐natural conditions in climatic chambers with 28/23°C temperature and 12/12 h day/night cycles (for details see Cremer & Heinze, 2003). Experimental colonies were checked daily for the eclosion of male pupae. Afterward, colonies were scanned five times per week until the death of the male.

Males were randomly distributed to three different mating groups, where we manipulated the number of available female sexuals. Wingless males were either kept virgin (V, *N *=* *46, without access to female sexuals) or given the opportunity to mate with a small number of female sexuals (low reproductive effort, LR, *N *=* *31, access to 1–3 female sexuals per week throughout their lives) or a large number of female sexuals (high reproductive effort, HR, *N *=* *38, access to 6–60 female sexuals per week throughout their lives).

To ensure that experimental colonies contained the appropriate number of virgin females, we added virgin female sexuals from nurse colonies, from which we regularly removed all male pupae. After emergence in these nurse colonies, virgin female sexuals were transferred to the respective experimental colonies. Data were collected in different years (2006: HR: *N *=* *6; V: *N *=* *10; 2007: HR: *N *=* *12, LR and V: *N *=* *10; 2014: HR: *N *=* *20, LR: *N *=* *21, V: *N *=* *26), but all colonies were kept under the same standardized conditions in the laboratory. Moreover, we verified that males of the same groups did not differ in their life span between the different years (C: *N* = 46; survival analysis χ² = 2.44, *p *=* *.29; LR: *N *=* *31; Cox′s *F*‐test: *F *=* *1.26, *p *=* *.29; HR: *N *=* *38; survival analysis χ² = 3.04, *p *=* *.22).

Initially, the subset of the 67 experimental colonies from 2014 was split into subgroups that were differently fed and kept either under limited (fed once per week) or ad libitum food conditions (fed three times per week), as trade‐offs were thought to become more obvious under resource limitation. Food availability did not influence male life span (ANOVA: *F*_1_ = 0.11, *p *=* *.74; interaction mating group × nutrition: *F *=* *1.80, *p *=* *.17), presumably because social insect workers direct scarce resources to the sexuals. We therefore pooled food‐restricted and ad libitum setups for subsequent analyses.

The life spans of males still alive at the end of the experiment were treated as censored (two HR males, one LR male). Likewise, male life span was censored in three colonies, which had to be terminated prematurely because of the emergence of a female sexual (2 V groups) or invasion by ants from another colony (LR group). In the 41 HR and LR colonies from 2014 and six HR colonies from 2006 (HR, *N *=* *26; LR, *N *=* *21), potentially mated young queens were removed after 5–7 days in the colony and subsequently dissected under a binocular to investigate whether they have been inseminated. If so, sperm was clearly visible inside the female sperm storage organ (spermatheca).

To check whether any differences in male life span might result from behavioral differences or different treatment by workers we set up additional colonies in 2014 (V, *N *=* *6; LR, *N *=* *7; HR, *N *=* *7) as described above and observed them for 3 min twice per day over 11 consecutive days. Depending on life span, we performed three to 21 observations per male. We recorded active (e.g., running, antennating, allogrooming, self‐grooming) and passive behavior (e.g., resting, being antennated or groomed) of the males and the male's location in the nest (center, periphery, outside).

All statistical analyses were performed in IBM SPSS Statistics (Version 21).

## 3 Results

Dissection showed that HR males mated with more female sexuals than LR males (HR: *N *=* *26, median [quartiles]: 11.5 [6.25; 23.5] female sexuals; LR: *N *=* *21, 3 [2; 5] female sexuals; Mann–Whitney *U*‐test: *U* = 84.5, *p *<* *.0001). Regardless of the mating group, males inseminated roughly three‐fourths of the available female sexuals (HR: median [quartiles]: 75% [58.3; 88.9], LR: 80% [75; 100]; *U* = 200, *p *=* *.12), perhaps because female sexuals sometimes refuse mating attempts. Laboratory colonies frequently contain several virgin queens despite the presence of males (unpubl. observations).

**Figure 1:**
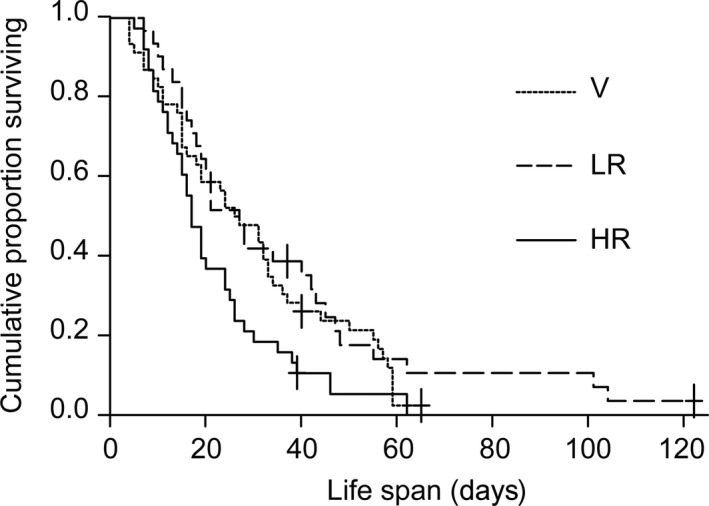
Life span (days) of *Cardiocondyla obscurior* ant males dependent on the availability of mating partner (V: no female sexuals available, LR: 1–3 female sexuals available per week; HR: 6–60 female sexuals available per week; censored data are indicated by cross‐hairs). HR males suffer a reduced life span relative to LR and V males

**Figure 2:**
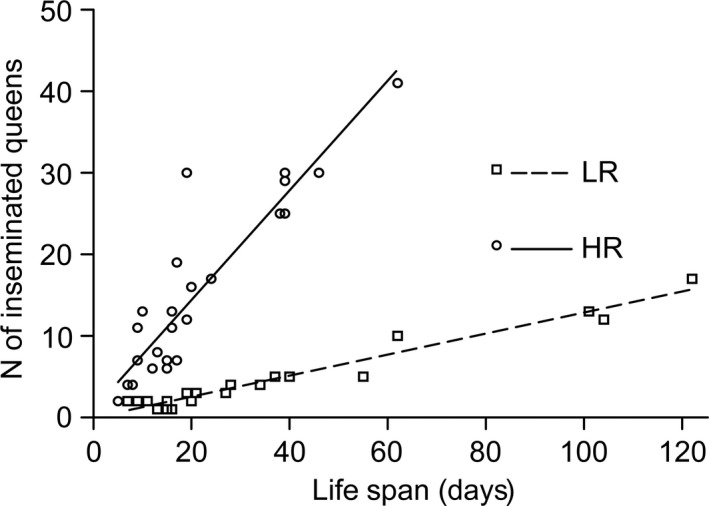
Life span and the number of females inseminated by males of the ant *Cardiocondyla obscurior* are positively correlated in both the LR and HR treatment

Males in the three mating groups differed significantly in life span (survival analysis log rank test: χ² = 7.67, *df *= 2, *p *=* *.022). Pairwise comparisons revealed that virgin males and LR males lived significantly longer than HR males (V: median [quartiles]: 26.5 [15; 40] days; LR: 27 [16.5; 44] days, HR: 17 [12; 26] days; HR vs. V: χ² = 4.03, *p *=* *.045; HR vs. LR: χ² = 6.67, *p *=* *.01; LR vs. V: χ² = 0.52, *p *=* *.47; Figure 1). The four most long‐lived males belonged to the LR group. In both groups, life span and number of inseminated female sexuals were significantly positively correlated (HR, *R* = .91, *p *<* *.0001; LR, *R* = .925, *p *<* *.0001; Figure 2).

Males of the different mating groups were similarly treated by workers and also did not differ in their location in the nest (all *p *≥* *.095). However, HR males tended to be more active than LR and V males (Kruskal–Wallis ANOVA: χ² = 5.52, *p *=* *.063; HR: 77% activity, LR: 46% activity, V: 48% activity).

## 4 Discussion

Our study shows that males of the ant *C. obscurior* that had access to a large numbers of female sexuals (HR) had a significantly reduced life span compared to males without (V) or with limited access (LR) to female sexuals. This indicates a fecundity/longevity trade‐off in HR males. Wingless *C. obscurior* males may encounter many receptive female sexuals during their lives (up to 50 or more, see (Heinze & Delabie, 2005)), and their lifelong spermatogenesis (Heinze & Hölldobler, 1993) enables them to mate frequently. Hence, HR males did not suffer unrealistic conditions in our experiment. Nevertheless, the pressure to quickly replenish sperm supplies and the elaborate courtship display (Mercier et al., 2007) may have negatively affected investment in body maintenance and repair and—in accordance with life history theory—results in the trade‐off between reproduction and longevity (Himuro & Fujisaki, 2010; Hou & Amunugama, 2015). Behavioral observations revealed in addition that HR males tended to be more active than V or LR males. The increased energy expenditure from moving might also have negatively affected life span, despite the fact that males have been probably not limited in resources, as no differences between food restriction and ad libitum food conditions were found. The costs of competition with other males, which shorten male life span in *Drosophila* (Bretman, Westmancoat, Gage, & Chapman, 2013), were excluded in our study by regularly removing all male pupae, but in unmanipulated colonies, wingless males of *C. obscurior* engage in lethal combat until only one male survives (Heinze & Hölldobler, 1993).

LR males appeared to have ample time between copulations for sperm replenishment, and their life span did not differ from that of virgin males. This suggests either that the costs of moderate levels of reproduction are very low or that infrequent mating has beneficial effects for the males that outweigh the costs of reproduction. The four most long‐lived males all belonged to the LR group, and the oldest LR male lived almost twice as long as virgin and HR males. Infrequent mating might therefore even prolong a male's life. Alternatively, staying virgin might infer some costs, for example, with regard to stress susceptibility or subtle changes in the behavior of workers toward males, which in the absence of mating partners are useless for the colony.

Our observation that LR males live as long or even longer than virgin males stands in striking contrast to data from solitary insects, in which virgin males outlive reproductive individuals (e.g., *Onthophagus binodis* dung beetles (Kotiaho & Simmons, 2003)) or in which male life span is negatively associated with mating frequency (*Anopheles* mosquitoes (Dao et al., 2010)), and also to data from other animals in which reproductively active males have a decreased life span (Hellriegel & Blanckenhorn, 2002; Nakatsuru & Kramer, 1982; Preston, Stevenson, Pemberton, & Wilson, 2001; Van Voorhies, 1992). However, it matches results from the only other study about the association between life span and reproductive efforts in social insect males: wingless males of the ant *Hypoponera opacior*, which mated at least once, lived longer than virgin males (Kureck, Nicolai, & Foitzik, 2013). Yet, this result might be attributable to early eclosing males having higher chances to mate than later eclosing males and workers killing *H. opacior* males after all female sexuals have eclosed (Foitzik, Heinze, Oberstadt, & Herbers, 2002; Kureck et al., 2013).

Despite the obvious costs of intense mating activities in *C. obscurior*, HR males had a considerably higher fitness despite their shortened life span. This again highlights the view that social insect males are characterized by a “live fast, die young” life history (Cappa, Beani, Cervo, Grozinger, & Manfredini, 2015; Heinze, 2016).

## Conflict of Interest

None declared.
